# When Minor Falls Are Overlooked in the Elderly: A Case Report of Vertebral Compression Fractures and Chronic Diaphragmatic Hernia

**DOI:** 10.7759/cureus.104527

**Published:** 2026-03-02

**Authors:** Nagi A Massoud, Abdulrahman H Alashkar

**Affiliations:** 1 Department of Surgery, Dr. Sulaiman Al Habib Medical Group, Buraidah, SAU

**Keywords:** dexa scan, diaphragmatic injury, fall injuries, geriatric, vertebral compression fracture

## Abstract

Falls are common in the geriatric population, and the high prevalence of osteoporosis in this population increases the risk of their associated injuries. Such injuries can be subtle and be overlooked by the patient or even physicians. In this case, we present a 78-year-old woman who presented following a fall with associated acute vertebral compression fracture (VCF) and was found to have another chronic VCF and a chronic diaphragmatic hernia that had been overlooked for over two years. This case shall highlight that physicians must be astute when assessing the frail elderly who can develop serious, yet subtle, injuries from what might appear minor trauma. Discovering such injuries helps not only in treating them early but also in implementing patient-specific preventive measures early.

## Introduction

Falls are a leading cause of injuries in the elderly. They can cause significant disability, especially as osteoporosis becomes increasingly more prevalent with aging [1,2]. Occasionally, falls may cause subtle injuries that could go unnoticed due to the absence of symptoms or the presence of only mild symptoms. Examples of such injuries, which the patient presented in this report sustained, are vertebral compression fractures (VCFs) (common) and diaphragmatic injuries (uncommon). Osteoporosis is the most common cause of VCFs [3]. In blunt trauma, the cause of diaphragmatic injury is a sudden increase in intra-abdominal pressure, which causes the diaphragm to rupture or tear due to the pressure gradient between the thoracic and abdominal cavities [4]. This case shall highlight that physicians must be astute when assessing the frail elderly who can develop serious, yet subtle, injuries from what might appear minor trauma. 

## Case presentation

A 78-year-old woman presented to our emergency department with mid-back pain one day following a fall down the stairs. The pain was well-localized, mechanical (aggravated by movement), and severe enough to impair her mobility. She did not have urinary symptoms, saddle numbness, or lower limb radicular symptoms. She denied having any chronic medical illnesses. However, she did report falling multiple times in the past and having "tolerable mid-back pain" that she had experienced for about two years following what she described as a "mild fall". Moreover, she reported chronic, yet worsening, heartburn, regurgitation, and nausea for which she had been prescribed a proton pump inhibitor. 

On examination, the patient was conscious and oriented and had normal vital signs. Lower limb examination showed no neurological deficits. Palpation of the spine showed no step deformity but was remarkable for mid-back tenderness. Her abdomen was soft and lax with no distention or tenderness. Her chest examination, however, was notable for the presence of rumbling noises on auscultation of the anterior and posterior chest walls at the mid-to-lower zones.

**Figure 1:**
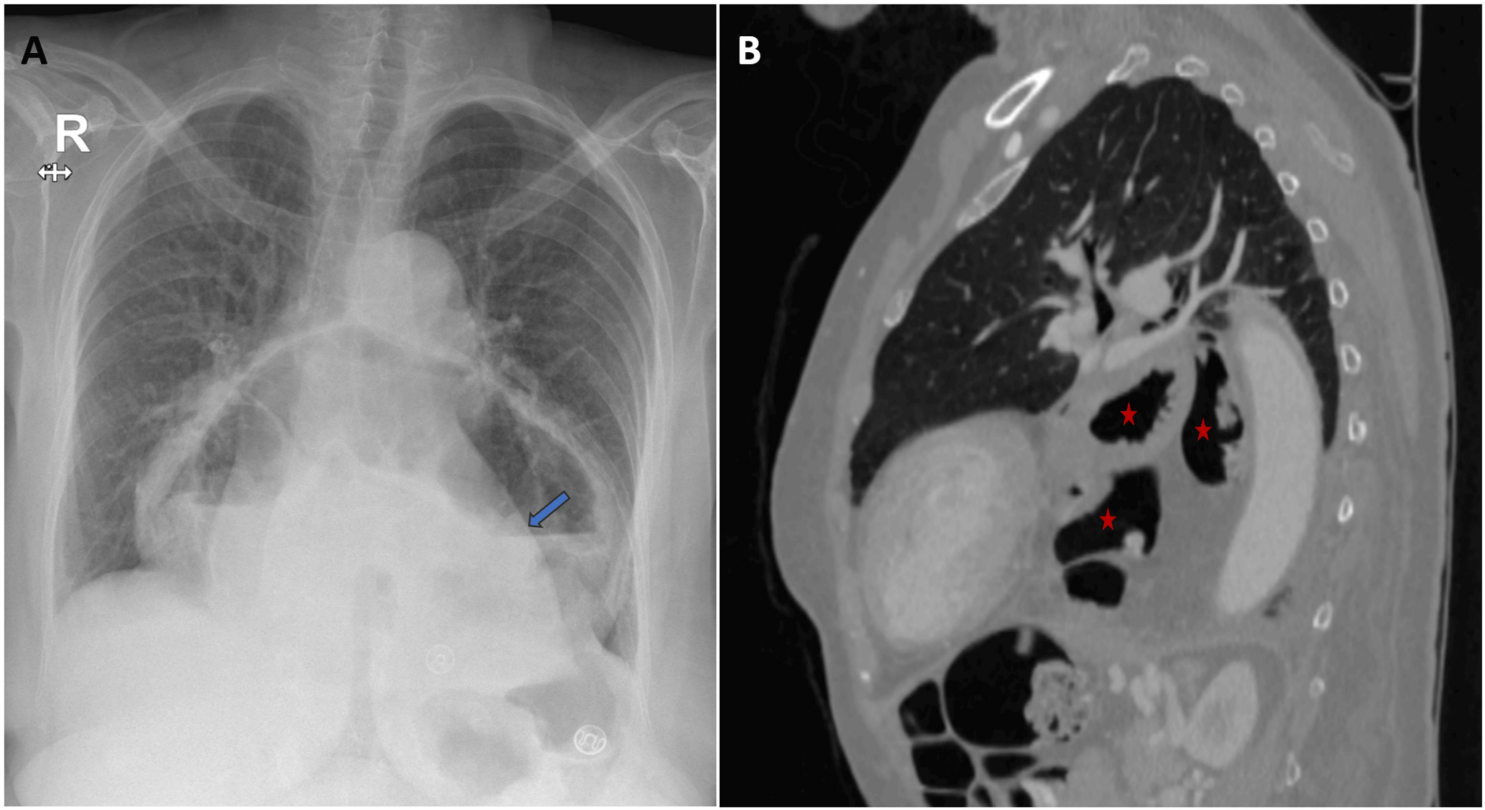
Left-sided diaphragmatic hernia Image A is a chest X-ray showing an air-fluid level overshadowing the cardiac silhouette (blue arrow). Image B is a sagittal CT image showing the presence of a left-sided diaphragmatic defect with the entire stomach, part of the transverse colon, and omental fat herniating into the thoracic cavity (red stars). CT: computed tomography

A chest radiograph revealed the presence of an air-fluid level overshadowing the cardiac silhouette, and computed tomography (CT) confirmed the presence of a left-sided diaphragmatic defect with the entire stomach, part of the transverse colon, and omental fat herniating into the thoracic cavity (Figure 1).

**Figure 2:**
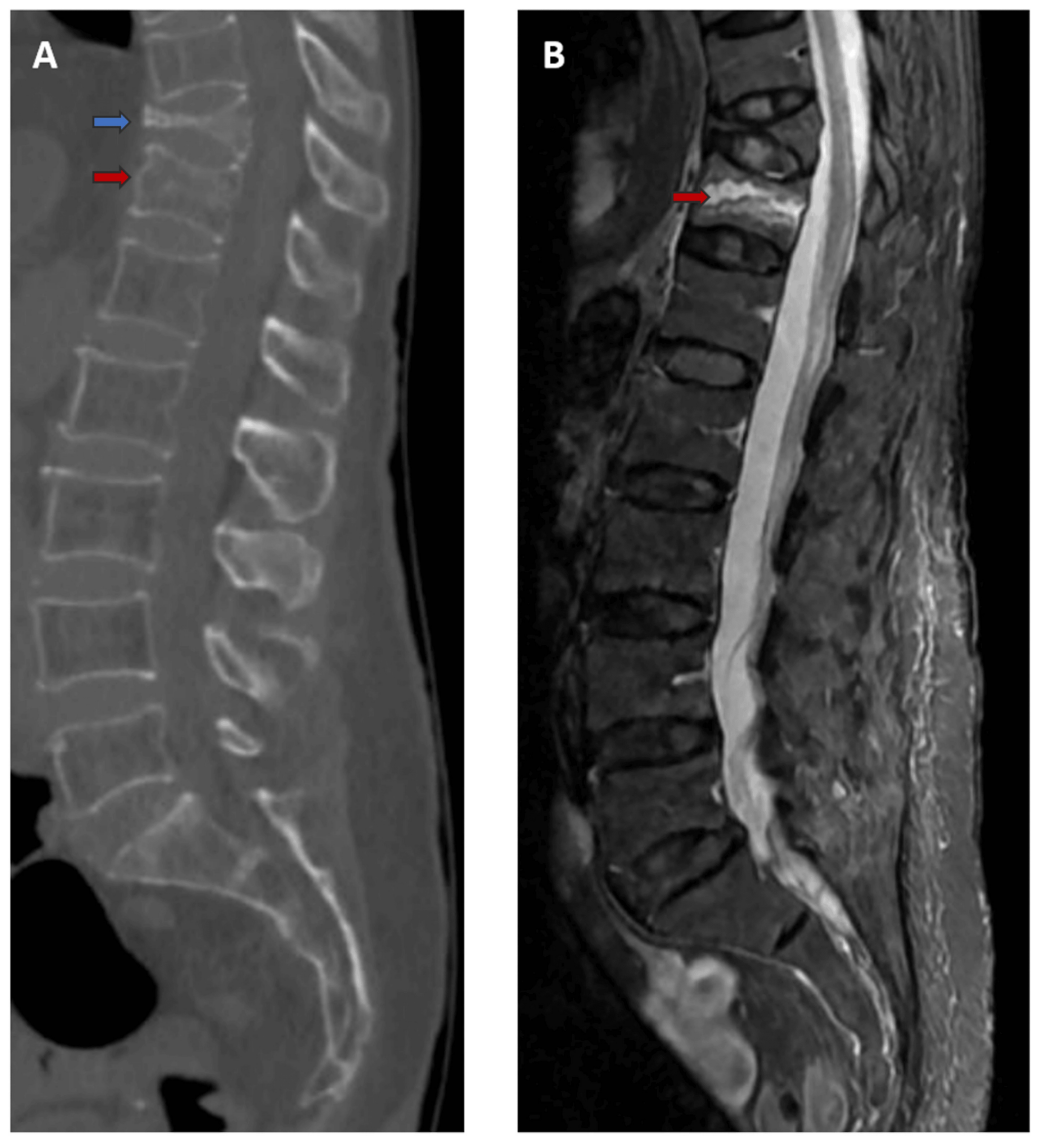
Vertebral compression fractures of the 11th (chronic) and 12th (acute) thoracic vertebrae Image A is a sagittal CT image showing compression fractures of the 11th (blue arrow) and 12th (red arrow) thoracic vertebrae. Image B is a STIR sequence MRI showing the same fractures and demonstrating the marrow edema (hyperintensity signal) of the 12th vertebral body (red arrow), which is indicative of acute injury. CT: computed tomography; MRI: magnetic resonance imaging; STIR: short tau inversion recovery

In addition, there were two VCFs involving the 11th and 12th thoracic vertebrae without spinal canal narrowing (Figure 2A). Magnetic resonance imaging (MRI) showed prominent marrow edema of the 12th vertebral body on short tau inversion recovery (STIR) sequence (Figure 2B). 

**Figure 3:**
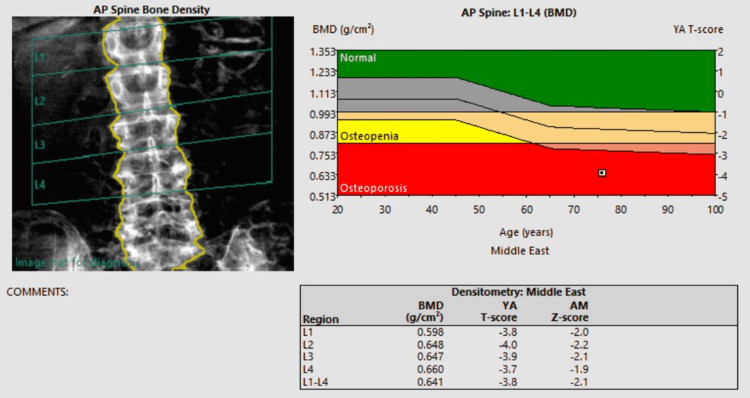
DEXA scan of the lumbar spine This image is the report of the patient's DEXA scan which shows her lumbar spine being in the osteoporotic range (T-score less than -2.5). DEXA: dual-energy X-ray absorptiometry; BMD: bone mineral density

Dual-energy X-ray absorptiometry (DEXA) confirmed a diagnosis of osteoporosis (Figure 3).

The patient was advised for conservative management (bracing and analgesia). However, after less than a week, she presented again with the same pain describing it as "intolerable with movement" and requesting an alternative treatment option. Following counselling and consenting, she underwent kyphoplasty of the 12th thoracic vertebra under local anesthesia.

Postoperatively, she reported immediate improvement in her pain. In her follow-up visit one week later, she was virtually pain-free. She was referred for further work-up and management of osteoporosis. Additionally, she was offered surgical repair of her diaphragmatic hernia, but she declined it.

## Discussion

Fall injuries cause significant morbidity and mortality in the geriatric population. It's estimated that about one-third of people over 65 years of age fall each year [1]. Multiple factors contribute to this large figure, such as decreased mobility, medical comorbidities (e.g., arthritis), and medication side effects [2]. Moreover, many of these patients are osteoporotic, which increases the risk associated with such injuries. An epidemiological analysis by Sadat-Ali et al. showed that 34% of women in Saudi Arabia aged 55-79 years old had osteoporosis [3]. This is reflected in this case where the patient had osteoporosis as demonstrated in her lumbar spine DEXA scan (Figure 3).

VCFs are the most common osteoporotic fractures. Their most common symptom is pain, which can range from mild and tolerable to severe and incapacitating. In addition, each VCF in the thoracic vertebrae impairs pulmonary function [5]. The usual first-line management of acute VCF without nerve or cord compression is conservative. This includes analgesia, bracing, and resuming mobility as soon as possible [6]. This was attempted with our patient. However, less than a week later, the patient requested an alternative treatment option due to persistent intolerable pain. She was offered kyphoplasty, which is a vertebral augmentation procedure. In a large, randomized trial that included 300 patients with acute vertebral fractures, kyphoplasty was found to be a safe and effective early treatment option [7]. Indeed, our patient reported immediate improvement in her pain.

In this case, and in addition to the acute VCF she presented with, she had a chronic one that had not been addressed earlier. Although the management of her first VCF would have been conservative, diagnosing it early could have led to the earlier treatment of her osteoporosis, which could have prevented subsequent fractures [8].

In addition to her chronic VCF, she had a chronic diaphragmatic hernia (CDH). Although the cause of the hernia couldn't be identified with certainty, her related symptoms of nausea, heartburn, and regurgitation can be chronologically correlated with the reported fall incidence two years prior to her current presentation.

Diaphragmatic injuries are uncommon, representing less than 1% of all traumatic injuries [4]. Moreover, they typically occur in conjunction with other injuries rather than being isolated. Also, about two-thirds of diaphragmatic injuries are secondary to penetrating trauma. When they do occur because of blunt trauma, the mechanism is a sudden increase in intra-abdominal pressure, which creates a large pressure gradient between the thoracic and abdominal cavities that causes the diaphragm to rupture/tear [4]. So, the occurrence of an isolated diaphragmatic injury secondary to minor blunt trauma is rare and unusual. Nevertheless, considering that she started to suffer from progressively worse symptoms (heartburn, nausea, and regurgitation) following a fall incidence, a diaphragmatic/hiatal hernia should have been kept in her differential diagnosis. However, this diagnosis was likely overlooked due to the absence of symptoms or the presence of only minor symptoms initially. Then, symptoms, such as heartburn and nausea, got worse over time as more abdominal viscera herniated into the thoracic cavity. 

Following the treatment of her acute vertebral injury, the patient was referred for further assessment and treatment of osteoporosis in order to prevent further fractures in the future. Also, the potential complications associated with the diaphragmatic hernia if left untreated (worsening symptoms and strangulation) were explained, and she was offered surgical treatment, but she declined.

## Conclusions

In the absence of nerve and/or cord compression (neurologic deficits), conservative management remains the first-line treatment of acute VCF. If conservative measures fail, vertebral augmentation needs to be considered, and kyphoplasty offers a safe and effective treatment option. Physicians must maintain a high index of suspicion for diaphragmatic injuries when new gastrointestinal symptoms, such as heartburn and nausea, develop following a fall, as early detection prevents chronic herniation and secondary complications. Also, physicians must be vigilant when assessing the frail elderly who can develop serious, yet subtle, injuries from what might appear minor trauma. Discovering such injuries helps not only in treating them early but also in implementing patient-specific preventive measures.
